# The Odontological Society of Great Britain

**Published:** 1883-08

**Authors:** 


THE
AMERICAN JOURNAL
OF
DENTAL SCIENCE.

VOL. XVII. THIRD SERIES—AUGUST, 1883. NO. 4.

ARTICLE I.

### THE ODONTOLOGICAL SOCIETY OF GREAT BRITAIN.

### DISCUSSION ON THE THEORY OF DENTAL CARIES.

At the February Meeting of this Society, Mr. Henry Sewill opened a discussion on "The Theory of Caries," and said that, as in any debate of this kind it was most important that the exact nature of the subject to be discussed should be clearly understood, he would commence by reading the question which had been decided upon by the Council to serve as a basis for the discussion. It was as follows:—"*Do the Incontrovertible Facts which we now possess as to its Etiology and Pathology fully account for the Phenomena of Dental Caries?*"

In his opinion there was no doubt that this question should be answered affirmatively. He believed that we now possessed a store of facts amply sufficient to account for all the essential phenomena of the disease. It was true that caries had been for many years successfully treated without any theory at all. It might, therefore, be asked by some what practicable good was to be expected of such a discussion as the present. But though the treatment which had been empirically followed had certainly been fairly successful, still there was no knowing but that it might be modified with advantage could we be certain of the true nature of the disease. Whilst as to the *prevention* of car ies, towards which little had been done as yet, this must depend almost entirely on our knowledge of its etiology and pathology.

Considering the importance of the subject—for caries was by far the most important of all dental diseases—it was remarkable that the question had remained so long unsettled. Even quite recently papers on this subject had been read before the Society based on somewhat astonishing physiological and pathological hypothesis. It had also been publicly stated by a member that no explanation could be given of the origin of caries; some even still asserted that it was a constitutional d sease, etc.

The first point to be decided was whether caries was due to external or internal causes. After that it might be possible to consider what were its exciting causes, and what its predisposing causes; what was the exact nature of the morbid changes in the tissue, and whether the tissues showed any vital reaction during the progress of the disease, or were merely passive.

He had no hesitation in saying that caries must be looked upon as a disintegration of tissue due to the action of *external causes.* The anatomical character of enamel and dentine appeared to negative the possibility of their being the starting point of pathological action; or, if such a thing was possible in dentine, it could not be possible in enamel, —a densely hard, almost homogeneous mass, containing a mere trace of organic matter, And it was in enamel that caries commenced, unless it started in a fissure. If caries commenced in these tissues as a true disease, we should certainly expect to find at an early stage some vascular disturbance of the pulp; but this never occurred. It was true that inflammation might occur in the cornea and other non-vascular tissues, but no gross lesion comparable to dental caries could take place even in the cornea, without vascular change. Nor were the cornea and dentine parallel cases; the one was soft and permeable, the other dense and hard. Dentine was, indeed, as a tissue unique.

It appeared to him there was one fact alone without those mentioned which effectually settled this question, viz., the fact that caries occurred in dead teeth and in artificial teeth made of ivory. It had been said that the vitality of the tooth would resist the action of external agencies. But though vitality might modify the process to some extent, causing pathological changes, it could not protect the tissues from the action of physical laws. A drop of nitric acid would destroy the skin, whether living or dead; and, similarly, acid would act upon a tooth whether living or dead.

What, then, were these external causes? One was acid. This was derived from the decomposition of food, from deranged secretions, acid mucus, etc. The minute phenomena of caries had lately been more fully cleared up by the investigations of Messrs. Underwood and Milles. They have found minute organisms flourishing in the organic matter of the dentine, and this accounted for the more rapid destruction of dentine than enamel. These organisms were of course introduced from without, and the chief point was that the whole process was due to the action of external causes.

Various objections to this view had been put forward, but these had all been satisfactorily explained. Thus it had been shown that the "translucent zone," which had at one time been thought to be a sign of "vital action," was equally well seen in dentine when softened by acid out of the mouth. So also with regard to the varicose condition of the dentinal fibres seen in carious teeth; these had also been shown to occur in dead teeth out of the mouth.

In the next place, what were the predisposing causes of caries? These might be shortly stated as anything which would be likely to render the enamel and dentine more easily acted on by acid; thus, fissures and other malformations of the enamel; soft porous enamel, and soft, badly formed dentine, containing interglobular spaces; crowding and irregularity of the teeth, favouring lodgment of decomposing *debris*, and interfering with proper cleanliness; and lastly, anything which tended to cause the formation of acid within the mouth, such as a bad state of the secretions, chronic dyspepsia, etc.

It had been asserted that the fact that the appearance of caries would sometimes date from an illness was a proof that the disease must be due to a constitutional state. But, in the first place, the change was not attended by any vascular phenomena, such as were met with in other lesions of constitutional origin, in whatever organ they might occur. And secondly, the condition of the mouth during many derangements of health, and many diseases, was eminently favorable to the existence of the local causes he had mentioned. In a case of typhoid fever, for instance, the teeth would be found covered with collections of vitiated mucus and partially decomposed food, which might remain for weeks; in pregnancy, as another instance in point, there was frequently a soft spongy state of the gums, with secretion of acid mucus, and often gastric derangement besides; in chronic dyspepsia the condition of things was somewhat similar,—unhealthy secretions, acid eructations, etc.

He did not pretend to speak as an authority who had made original investigations on this subject. He was only expressing his own individual opinions. But it did appear to him that the evidence in favor of the local origin of caries, and that it consisted of disintegration of the tissues without pathological phenomena of any kind, was almost overwhelming. He thought it was impossible for any one who read carefully through the masterly summary which was given in Messrs. Tome's work, based on original research, to continue in doubt on this question. The conclusion arrived at by these authors was that caries had, properly speaking, no pathology.

The well-known work of Wedl also gave a most philosophical and judicial review, based on original investigation, of all that was known on this subject. He described dental caries under the head of "Anomalies of the Secretions," and decided strongly against the existence of any inflammatory origin, or of any vital reaction in the tissues during the progress of the disease.

Leber and Rottenstein also furnished important corroborative evidence, though they were mistaken in ascribing to the action of leptothryx what was no doubt due to the action of bacteria and other organisms. Magitot also had, by a careful series of experiments, shown that enamel and dentine were destructible in acids not stronger than those formed in the mouth. So also Westcott, Allport, Mantegazza, and last but not least, Messrs. Underwood and Milles, had all produced important evidence to show that caries was due to ordinary physical causes acting from without.

In his desire to be brief, he had left out all reference to several most important points bearing on this question; but he knew there were other members ready to take part in the discussion, and that some of them were more competent to deal with these matters than himself. He hoped that the Society would be willing to discuss the question thoroughly, since this might possibly have the very desirable result of removing, to a considerable extent, the state of uncertainty in which it had so long remained.

Mr. A. Coleman said Mr. Sewill had started with the proposition that, inasmuch as dental caries commenced on the surface, and was due entirely to external causes, it was not a disease. But if they accepted that definition, they would have to exclude from the category of diseases many morbid conditions which had hitherto been included within it. Thus, some of the skin diseases, ringworm and favus, for instance, commenced on the surface, and were due entirely to external causes. It was true that in the progress of these diseases inflammatory conditions were manifested, and that such conditions had not been clearly demonstrated in dentine during the progress of dental caries. It was hardly to be expected that such manifestations could be made apparent in a structure of such a character, but the fact must not be ignored that some investigators maintained that such changes could be shown. Putting aside the disputed nature of the "translucent zone,"—claimed, however, by Magitot, a strong advocate of the chemical theory of caries, as an evidence of vital action in the dentine,—there were the researches of Dr. Frank Abbott, who demonstrated that, in the enlarged canaliculi, granules and threads exist which took up carmine, whilst in parts further affected these were filled with partly-nucleated protoplasm. These phenomena might of course be explained as indicating the presence of organisms, originating from without, and which had found their way into the canaliculi. But if this were so, what could be said in the case of caries occurring in cementum? He thought it could hardly be denied that we had found the same evidences of inflammatory action, shown by the presence of corpuscles, as appeared in bone during the progress of inflammation.

Mr. Sewill stated, with considerable confidence, that precisely the same conditions occur in the decay of dead teeth as in that of living teeth. Now this was a point upon which all were not agreed. Dr. Frank Abbott stated distinctly that the appearances presented under the microscope were not the same in the two cases; and he (Mr. Coleman) would also say that, in his opinion, there were differences in the general appearance and conditions met with in each. In the case of teeth attached to artificial dentures, the softening of the dentine was more general and superficial, and the enamel was more friable than in the case of living teeth affected by caries. It was also his opinion, founded on long observation, that dead teeth and teeth that had their pulps devitalized, when attacked by decay, ran a more rapid course, and that the softening was less circumscribed than in teeth with living pulps.

That the appearances presented by decay in dead teeth and the condition termed caries in living teeth, should, in the parts actually affected, present very similar appearances, was what the advocates of a vital theory would strongly maintain. Those who believed that a healthy living tooth possessed an inherent power, which protected it from external chemical influences, would admit that a tooth attacked by caries had lost that power, or that it had been overcome in that part which had become affected by the disease.

With regard to the external influences which might, directly or indirectly, determine caries, an acid state of the secretions of the mouth was no doubt an important factor. But if it were the sole cause, would not the result be a more general action upon the teeth? as had, indeed, been pointed out by Hunter. And then, with regard to the evidence of this acid state of the saliva, he had himself, many years ago, made a very large number of observations upon this point, expecting to be able to connect an acid condition of the saliva with, at all events, very active or acute cases of caries. But he obtained no such result. In cases of pregnancy and those convalescent from the exanthemata, the conclusion was the same, and yet in such cases it often occurred that teeth which, until these conditions supervened, had been excellent, then underwent very rapid decay. This had led him to doubt very much a simply acid cause for caries, and had brought him to believe that the greater liability to disease in such cases arose from a lowering in the power of resistance of the teeth to agencies external to them,—that is to say, to the ordinary laws of chemical affinity.

The usual hour for adjournment having arrived, the President suggested that, as no doubt there were many other members anxious to state their views on this question, it would be a pity to attempt to finish the discussion that evening, and he would put it to the meeting that it be adjourned. This was agreed to.

The discussion was resumed on the 4th of June, which was the last meeting of the Society during the present session. On this occasion, the president, in opening the adjourned debate, thought all would admit that dental caries was, to some extent, due to external causes—to the solvent action of acids generated in the mouth by fermentation. But he could not think that this was the only, or even the chief cause. If it were, how could the fact be explained that of two adjacent teeth exposed to the action of the same acids, caries might be arrested in one, whilst its neighbor would continue to crumble away? Moreover, the presence of these acids in the oral cavity was not a new condition of things; hence he thought the chief cause of the present increase of caries must be looked for in the teeth themselves, and, most probably, consisted in defective development. Dr. Hitchcock assured them that among the emigrants in America the teeth deteriorated after two generations. This defective development might be due to deficiency of mineral salts in the food, either in the quality of the water supply, or from deficiency of fresh vegetables, or the removal of the husk from cereal food; or it might arise from undue excitement and irritation of the nerve centres during the development of the dental tissues. Whilst Mr. Sewill deserved their thanks for the way in which he had brought forward certain facts which had been established by the labors of many distinguished observers in this country, on the Continent, and in America, he (the President) thought sufficient prominence had not been given to some other facts which were of equal, if not greater, importance in this connection.

Mr. W. J. Milles said he had been asked to say a few words on the subject under discussion, and he should confine his observations more particularly to the points which Mr. Underwood and himself had been studying together, viz., the action of micro-organisms in the production of dental caries. They found in all cases of caries micro-organisms were present in certain definite lines. In the most superficial part of caries the tubes were broken up, and were oval in shape. In deeper parts the organisms became less numerous, and in the most internal parts they were in single file. These observations, which they had published nearly two years ago, had been confirmed in various ways. Dr. Miller, of Berlin, had recently substantiated them in nearly every point, though he had made some different deductions. But whilst he considered that the micro-organisms had little to do with the active phenomena of caries, he thought this was essentially a process of decalcification due to acids produced by fermentation in the mouth. It was known that fermentation elsewhere was always due to the presence of micro-organisms, and no doubt fermentation in the mouth was due to the same cause. Some support was given to this view of the active participation of micro-organisms in the production of caries by the recent discoveries by Pasteur of the active part they take in the production of other diseases. In some of the diseases of animals the agency of micro-organisms had been scientifically proved, as in the case of anthrax and chicken cholera ; and the discovery of the bacillus of tubercle seemed to indicate that a large number of human diseases were also thus caused. Unfortunately they had not yet been able to produce true caries out of the mouth, but this, no doubt, arose from the impossibility of reproducing artificially all the conditions which were present in the mouth. Very great difficulty had been met with in the artificial cultivation of other special organisms, as, for instance, the bacillus of tubercle ; each special organism required certain special conditions of life, and what those conditions were in the case of the micro-organisms of caries had not yet been discovered. There was, however, the fact that changes precisely similar to those which occur in the caries of natural teeth, had been found to occur also in the ivory dentures which were formerly used in Dentistry, the conditions here being similar. The fact, therefore, that caries could not be made to go on in teeth which have been removed from the mouth was not opposed to the view that micrococci and bacteria were really the active causes in its production, since this, no doubt, was due to the fact that we could not maintain, out of the mouth, exactly the conditions necessary for their normal production.

Mr. Chas. Tomes said he had unfortunately not been able to make any recent observations of his own on the subject of dental caries; but so far as his own slight investigations into the subjnct went, he was inclined to agree with the conclusions of Messrs. Underwood and Milles, though there was a good deal yet to be made out. It was clearly established that micro-organisms do accompany caries, and that they do so in a certain way; it was known also that micro-organisms which are capable of cultivation breed true, that each kind produces its like, and that they do not run into one another, as Dr. Miller supposed. In Dr. Miller's Paper there were investigations of inoculation into both sound and unsound dentine, which were very valuable, showing that micro-organisms were capable of growing upon dentine, and that is was possible for them to be growing, and yet all the conditions for producing caries were not present. It was certain that there were a number of other influences required for the production of caries. Thus he had himself met with several patients who, having had exceptionally sound teeth up to full adult life, had then suffered from a severe attack of typhoid fever, after which caries had appeared, and progressed with most extraordinary rapidity, all round the necks and in between the teeth in all directions. Now, it was not likely that these mouths had escaped infection with micro-organisms up to that time, but probably the disordered secretions left behind by the typhoid fever had afforded to the micro-organisms the pabulum needed, not only for their own growth, but also for the development of those acids which decalcify dentine and perform an important part in the production of caries. A single carious tooth in the mouth must supply micro-organisms enough to destroy every other tooth, and the reason why they did not destroy them could only be that some of the co-incident conditions necessary were not present, and probably these were most often found in the state of the oral secretions. For he found that altogether he could produce a temporary multiplication of the micro-organisms under certain conditions, still they appeared to be incapable of attacking the sound dentine, some of the conditions necessary to enable them to do this being evidently absent.

Dr. W. St. George Elliott, agreeing with Mr. Chas. Tomes as to the uselessness of anyone stating merely general impressions without recording observations and facts, remarked that the idea that micro-organisms were the cause of dental caries was a very old one. In old books toothache was always said to be due to worms gnawing at the nerve, and the same belief was general at the present day throughout the less civilized nations of the world, even in parts as far asunder as China and Japan and South America, where cure was supposed to follow the extraction of these worms. He had had the opportunity of seeing a very large number of Dr. Miller's preparations, probably over a thousand of them, and they certainly appeared to bear out the statements which had been made with regard to them. At all events he (Dr. Elliott) could assure them that Dr. Miller's experiments were honestly made, that he had no hobby to bring forward, his sole idea was to demonstrate the truth, and he certainly deserved credit for his work. In surveying the different theories of dental caries it appeared to him that it was impossible to accept any one as a complete explanation; the necessities of the case could only be met by combining together two or more of those which had been put forward. The chemical theory was the most popular at the present day, and it accounted for a good deal. But if chemical action was invariably the cause of decay, how was it that caries did not always occur at the points most favorable for their action, *i e.*, between the teeth; but caries might occur at any point of the mouth, and at any part of the teeth. Of course it was readily answered that there must also exist a defect tn the tooth at the point thus acted upon, and he thought that the significance of defective development and the chemical action of acids was now generally recognized

Mr. Moon said he had only general impressions to offer, but these lead him to think that overcrowding had a good deal to do wiih the production of decay. When teeth were closely packed together the attrition they exercised on each other might be sufficient to damage the enamel, and thus let in the active agents of decay. If you examine old skulls you will find the sides of teeth which have been closely in contact very often present polished facets ; and if in these strong old teeth we get polished facets, in weak modern ones we are likely to get sufficient derangement of enamel to let in the active agents of decay. It is a constant experience that young people come to us, and with the exception of the lower incisors, every tooth in their head is decayed whenever its touches it neighbor. Besides the weakness of modern teeth there was probably also some unhealthy condition of the oral fluids which was not previously present. The comparative immunity from decay of the lower front teeth was worthy of consideration. His experience was that the lower front incisors and canines escaped decay wonderfully as compared with other teeth until a lower artificial denture was inserted. These teeth might escape because they received a constant alkaline wash from the saliva poured out by the sub-lingual glands. He fully concurred in the remarks made at a recent meeting of the Society with reference to the desirability of great attention being paid to the diet of children in infancy, and to the diet of pregnant mothers. He thought this aspect of the question was of national importance.

Mr. S. J. Hutchinson thought that there was a tendency to wander from the question before them. They were not asked to give their own theories of the production of caries, they were simply asked, " Do the incontrovertible facts which we now possess as to its etiology and pathology fully account for the phenomena of dental caries ? " He asserted that the " incontrovertible facts," upon which Mr. Sewill appeared to rely, were not all of them incontrovertible, and on the whole he was inclined to support the view taken by Mr. Coleman at the February meeting rather than that taken by Mr. Sewill. His own opinion was that dental caries was not entirely due to external causes, but that there was a vital element in the process. Mr. Sewill had compared the dentine with another non-vascular tissue, the cornea of the eye; he admitted that inflammation might occur in the cornea, but would not allow that it could occur in dentine. "The one," he said, "was soft and permeable, the other dense and hard." Now it appeared to him (Mr. Hutchinson) that the structure of the dentine made it exceedingly probable that some action was constantly going on through its soft organic fibres, just as a certain circulatory process went on through the intercellular spaces of the cornea. It would be in the experience of all that the substance of the six-year-old molars became harder as the child grew up to adult life, and this must be due to some vital process. Again, it was well known that when a tooth was attacked by caries a secondary deposit of dentine would often take place opposite the deposit of a commencing decay. Must not this be due to some influence circulating through the fibres of the dentine, by which the action which was going on at the suface of the dentine was communicated to the periphery of the pulp? Mr. Hutchinson, in conclusion, read the following extract from Dr. Norman Kingsley's Paper, read at the International Congress, which, he said, fully expressed his views: "The teeth require as much constant nutrition as the muscles, the bones, or any other organs or tissues of the body. Teeth decay, primarily, because the nutrition of their organic structures being withdrawn, retrograde metamorphosis ensues. . . . Caries is simply solution or disorganization of tooth constituents by agents which are always external, but which would be quite inert under other constitutional conditions. The vitality of well organized tooth structure in a healthy body is quite sufficient to counteract the effects of any active external agents which might be temporarily present; but when nutrition is insufficient or diverted, the resisting power of its vitality inadequate, and destructive agents present, the teeth will yield at their weakest points and caries will result."

Mr. Charters White said he entertained a strong view that dental caries was mainly due to chemical action. It would generally be found that caries manifested itself in places where a lodgment could be formed, for instance, in the interstices of the teeth and in pits and fissures on the grinding surfaces. So in the case of patients who wore an artificial denture and were not very cleanly in their habits, you would find the pattern of the plate marked out on the teeth all round with superficial caries and decalcification. But if patients kept the insides of their plates perfectly clean and polished no disease would occur, showing that it was entirely due to chemical action. Still he did not think that vital influences could be altogether excluded. Vital action might not have much to do with the production of caries, but its existence could be recognized in its attempts to oppose the progress of decay; thus when caries was approaching the pulp cavity it was common to find a nodule of dentine inside the cavity which served as a shield to keep it out. For this and other reasons he thought the existence of vital action in the dentine could not be denied, although chemical action, no doubt, played a great part in the matter.

Mr. Coleman said that as he had taken up so much time at the previous discussion, he would only beg permission to make a few brief remarks and call attention to a point which he had omitted to mention on the previous occasion. Was it not a fact that in cases of caries, especially of the rapid or acute form, the exposed dentine became extremely sensitive? It was possible to drill into healthy dentine, as in some of the methods of pivoting now practised, without causing much pain, but in these cases the dentine eventually became acutely sensitive; he thought that this afforded some evidence of a pathological change. He thought all would admit that external agencies were the primary cause of dental caries. But these acids, fermentative processes, etc., were continually acting; the teeth were constantly exposed to them, more or less. Even with regard to the fissures that had been spoken of, they were sometimes the starting point of caries; but sometimes they might exist throughout life, and no decay take place. Therefore he thought that where decay did take place, there must be a failure—a want of something which arrested and counteracted the action of those principles that were constantly present and ever ready to act upon the teeth.

Mr. Stocken thought that the phenomena of caries could be accounted for by chemical action, favored in the majority of cases by structural defects in the teeth; Dr. Field was of opinion that dental caries was due partially to chemical causes, and partly to the action of micro-organisms.

Mr. Sewill, in reply, said the opinion he had endeavored to express in his opening speech was that caries was caused by external agents, that the dental tissues were perfectly passive under these agents, and that there was no pathological action; and to this opinion he was still prepared to adhere. The discussion that evening had gone off into details as to the exact changes which take place in the tissues, which were not of great importance, and as to which more would hereafter be made out. The point of importance was to disprove the statements made that evening, that the teeth act and react from constitutional causes. He maintained that our knowledge of the structure of dentine and enamel, and our knowledge of anatomy and physiology, justified us in stating that it was impossible that these tissues could be the seat of anything which could be compared to the pathological action that might occur in any other tissue of the body. Mr. Coleman had adopted the view of Dr. Abbott, who imagined that he could see in carious dentine the phenomena presented by inflammation in other non-vascular structures, and who depicted corpuscles and other inflammatory products in the dentine. He suggested that Mr. Coleman should explain how inflammatory corpuscles could get into the substance of dentine—a calcified matrix permeated by tubes not more than 1-4500 of an inch in diameter, and which were occupied by fibrillæ. The facts mentioned by Mr. Coleman with regard to cementum did not help the question; cementum was bone, and it was not astonishing that inflammation might occur in it under favorable circumstances. With regard to the irritation of the pulp being a sign of vital action, he thought that it was a simpler explanation to suppose that it was due to the fact that the partial destruction of the dentine rendered the pulp more exposed to sudden changes of temperature, and it was no doubt also partly due to the presence of the bacteria, which were known to penetrate along the tubules. He thought it was an "incontrovertible fact" that dental caries, identical with that which occurred in living teeth, might occur in dead teeth, and also in hippopotamus ivory. This one fact appeared to him sufficient to establish his case. It was quite unnecessary to imagine this mysterious "vital influence," and they must reject such a theory. All the phenomena of caries could be readily explained by reference to physical and chemical causes, and this view was more in accordance with what was known respecting the structure and mode of development of enamel and dentine, to say nothing of the support which it received from the eminent authorities whose opinions he had quoted in his opening remarks. He hoped that the subject, in some form or other, would again be brought before the Society.

### CASUAL COMMUNICATIONS.

At the meeting on the 4th ult. the Curator showed for Mr. G. Holt Bury, a model of the upper jaw of a boy, ten years of age, which showed the presence of four conical incisors and two small molars. The lower jaw was quite edentalous. The upper incisors appeared at about the age of four years, but the eruption of the molars was not noticed. The child was very delicate and had white hair. Other children of the same family had normal teeth.

Mr. Hutchinson also showed a model of two supernumerary teeth in the central region of the upper jaw, the case having been sent by Mr. Richards, Hastings.

Mr. Stocken showed a model of the upper jaw of a child nearly five years of age, with two abnormal teeth in the front of the mouth which were erupted at about the age of two years. The lower jaw was edentalous. The child was intelligent, had a large head, and never perspired through the skin. The child was nursed for ten weeks, but in consequence of the mother having abscess of the breast, it afterwards had to be fed upon milk and Dr. Ridge's food.

Mr. Coleman was inclined to think that the teeth were permanent in the case mentioned by Mr. Stocken. They seemed to resemble cases of eruption of the teeth at a very early age lately brought before the Society, and he thought that the two teeth must have had temporary predecessors.

Mr. Stocken had made inquiry, and was assured that there had not been any predecessors.

Mr. W. H. Coffin showed a contrivance, consisting of a brass tube about an inch in diameter, filled with very small glass beads, and having a diaphragm of copper guaze at each end to keep them from coming out. It was designed to silence the noise of liquid nitrous oxide gas rushing from the bottle to the bag, which was so alarming to nervous patients; and also to save the bursting of the bags from the sudden expansion of gas, in cases where the operator used the gas direct from the bottle to the bag. Mr. Coffin demonstrated, with very great success, the use of the tube, and remarked that the invention was patented by Mr. Justice, in Philadelphia, and was used largely in torpedo launches, were silence was indispensable, and also in exhausting steam from large boilers. He (Mr. Coffin) had adapted the principle to the gas bottle, and had obtained the permission of the inventor to manufacture one an exhibit it. Mr. Justice also intended manufacturing them and supply the dealers at a reasonable price, so that members of the Profession would be able to obtain them at a small cost from the Dental Depots. Mr. Coffin also showed the foot key he used for turning on the gas bottles.

Mr. Coleman mentioned a case from Mr. Read, Finsbury Square, who, in extracting a tooth, brought away part of the floor of the antrum.—*Dental Record.*

